# Smartphone Apps in the Context of Tinnitus: Systematic Review

**DOI:** 10.3390/s20061725

**Published:** 2020-03-19

**Authors:** Muntazir Mehdi, Constanze Riha, Patrick Neff, Albi Dode, Rüdiger Pryss, Winfried Schlee, Manfred Reichert, Franz J. Hauck

**Affiliations:** 1 Institute of Distributed Systems, Ulm University, 89081 Ulm, Germany; 2 Department of Psychology, University of Zürich, Box 1, CH-8050 Zürich, Switzerland; constanze.riha@uzh.ch; 3 Clinic and Polyclinic for Psychiatry and Psychotherapy, 93053 Regensburg, Germany; patrick.neff@uzh.ch (P.N.); winfried.schlee@tinnitusresearch.org (W.S.); 4 URPP Dynamics of Healthy Aging, University of Zürich, Box 2, CH-8050 Zürich, Switzerland; 5 Institute of Databases and Information Systems, Ulm University, 89081 Ulm, Germany; albi.dode@uni-ulm.de (A.D.); manfred.reichert@uni-ulm.de (M.R.); ruediger.pryss@uni-wuerzburg.de (R.P.)

**Keywords:** mobile health, smartphone apps, tinnitus research, biomedical and health informatics

## Abstract

Smartphones containing sophisticated high-end hardware and offering high computational capabilities at extremely manageable costs have become mainstream and an integral part of users’ lives. Widespread adoption of smartphone devices has encouraged the development of many smartphone applications, resulting in a well-established ecosystem, which is easily discoverable and accessible via respective marketplaces of differing mobile platforms. These smartphone applications are no longer exclusively limited to entertainment purposes but are increasingly established in the scientific and medical field. In the context of tinnitus, the ringing in the ear, these smartphone apps range from relief, management, self-help, all the way to interfacing external sensors to better understand the phenomenon. In this paper, we aim to bring forth the smartphone applications in and around tinnitus. Based on the PRISMA guidelines, we systematically analyze and investigate the current state of smartphone apps, that are directly applied in the context of tinnitus. In particular, we explore Google Scholar, CiteSeerX, Microsoft Academics, Semantic Scholar for the identification of scientific contributions. Additionally, we search and explore Google’s Play and Apple’s App Stores to identify relevant smartphone apps and their respective properties. This review work gives (1) an up-to-date overview of existing apps, and (2) lists and discusses scientific literature pertaining to the smartphone apps used within the context of tinnitus.

## 1. Introduction

Tinnitus is a complex and heterogeneous disorder associated with causing the perception of a continuous clicking, ringing, roaring, or buzzing sound (noise) in the ears in absence of any external sound source. Approximately 15% of the world’s population suffers from tinnitus, wherein 2% of these experience a substantial decrease in quality of life due to the phantom percept [1]. Many factors associated with causing this phantom sound are still unknown, yet, it is often associated with an underlying damage in the ear, such as the loss of cochlear hair cells. The loss of the hair cells can have different origins: a common risk factor is an acoustic trauma (exposure to loud sounds), the same applies to ototoxic drugs. However, tinnitus can also develop as a symptom of a cochlear affecting disease, such as Ménière’s disease (MD), or in the course of aging and age-related hearing loss (presbycusis) [2,3]. Age-related physiologic changes, for example, degeneration of sensory receptor cells, are one common cause of disorders of the sensory systems, like the auditory system [4]. Further age-related changes have been identified in auditory processing in the brain and may be related to the generation of dementia [5,6]. Besides the increased risk of tinnitus with higher age, elder persons have also been shown to experience more tinnitus-related distress which is theorized to be related to decreased compensatory brain plasticity [7]. In a steadily aging society, presbycusis and tinnitus thus become more prevalent with consequences beyond auditory sensory handicaps.

Presently, tinnitus is considered as a condition that involves changes at different levels of the auditory pathway, the auditory cortex as well as non-auditory areas like the limbic system. These changes may additionally be influenced by psycho-social stress (for example, negative thoughts, the argument at home, increased workload, etc.), affecting the emotional status and the auditory system [8,9]. Consequently, variations in tinnitus loudness and tinnitus-related distress, as well as the individual perception of tinnitus has been often reported by tinnitus patients [10]. Additionally, tinnitus variations can be directly or indirectly affected by changes in the atmospheric surrounding [11] and environmental conditions of the patient [12]. Individual case studies on weather conditions and their impact on fluctuations in tinnitus show limited but some evidence of a connection [13,14]. In patients suffering from MD [15], which commonly occurs with hyperacusis [16,17], a weather change usually contributes to tinnitus increase [18]. Abrupt change in barometric pressure (particularly reduced pressure) may cause or increase tinnitus symptoms because it affects the eardrum, the round window, and the cochlear fluids. Increased wind speed or humidity also worsen the tinnitus symptoms due to influences of high sensitivity on the ears [12]. A similar relationship applies to seasonal change [19].

Smartphone-based Ecological Momentary Assessments (EMA) methods can be utilized to capture the variations in tinnitus perception and link them to current surrounding or environmental conditions of the patient [20]. Furthermore, the tinnitus variations related to stress can be coped with using smartphone-based Cognitive Behavioural Therapy (CBT) or self-help apps, and individual perception of tinnitus can be managed using smartphone-based tinnitus relief apps. Despite smartphones, smartphone apps, and auxiliary health devices, for instance, heart meters, activity trackers, and smart wristbands, have become popular in assisting patients in managing and controlling their health problems [21,22], further research to determine the effectiveness of these applications and devices in different domains of healthcare is still required [23,24]. Nonetheless, smartphones are interesting in particular as most of today’s smartphones provide high computational power, a long-lasting battery life, and incorporate a set of sophisticated built-in sensors that are capable of accurately monitoring environmental surroundings and can be programmed and managed by apps. Additionally, smartphones provide an application ecosystem, extendable to program and include new apps targeting different health problems at almost negligible costs. New smartphone apps can be designed or existing apps can be tailored to assist in managing or mitigating the symptoms of different health problems [25]. For instance, mobile crowdsensing and smartphone-app solutions can be applied to monitor the ecological or environmental surroundings of patients using the built-in sensors [26,27]. Similarly, for tinnitus, these smartphone-app–based solutions also apply. However, due to the fast-growing development and the continuous publishing and inclusion of new apps in the app market places, the current state of smartphone apps within the context of tinnitus is mostly unbeknownst to patients and clinicians alike.

In this paper, based on the PRISMA guidelines [28], we explore online scientific literature sources namely: Google Scholar, CiteSeerX, Microsoft Academics, and Semantic Scholar as well as app stores, namely: Google’s Play Store and Apple’s App Store to list and identify tinnitus-related smartphone apps. The idea of this paper is to list and index smartphone-based solutions for assisting patients suffering from tinnitus, to foster a better understanding, management and treatment (by the provision of therapeutic solutions), as well as monitoring the severity of their tinnitus. Likewise, we report on apps that succor tinnitus patients in testing for hearing impairment (usually accompanied by tinnitus [29]), and, if possible, protect and train the remaining hearing abilities.

A review by Sereda et al. [30] lists tinnitus management apps based on patient opinions, gathered via a web-based survey. Moreover, the apps identified through a web-based patient survey are further evaluated based on the Mobile Application Ratings Scale (MARS) [31]. The added value from our review is primarily the exploration of the smartphone app markets to reveal relevant apps, as opposed to using a survey. The review by Kalle et al. [32] discusses internet- or smartphone-delivered CBT, with particular focus on self-help for tinnitus. The authors demonstrate the role of several approaches in advancing tinnitus clinical practice, but have focused less on current and available apps for patients. The review by Lui et al. [33] addresses efficacy or effectiveness of mental-health-app–based therapeutic solutions, but not with a particular focus on tinnitus. However, they do consider apps based on CBT, one of the most common therapies in the tinnitus domain. In our review, we do not limit the scope to CBT, self-help, or mindfulness apps, rather we expand further to address apps that also fall into the non-therapeutic category. In another article [34], the authors have outlined hearing healthcare apps from prominent smartphone platforms. However, the list of apps is limited and most apps have been outdated. Similarly, Bright and Pallawela [35] discuss smartphone apps for hearing assessments including comparison and validation of apps. In comparison, the scope of our proposed work is not limited to hearing assessments, but further includes additional apps for hearing healthcare, for instance, hearing protection and enhancement apps.

In summary, unlike the aforementioned studies and reviews, the objective of the presented review is to identify and report on smartphone-based solutions (apps specifically), within the context of tinnitus, that are, in turn, widely and easily available on mainstream app stores. Additionally, a further objective is to report on the current state of smartphone-based app solutions presented in the literature, be that either in the form of discussing the underlying technology or technique used for the development of the smartphone app, or the effectiveness of the smartphone apps for tinnitus patients. The overall process of identification of smartphone apps on scientific literature sources as well as on app store markets is detailed in Section 2. The identified results are reported and discussed in Section 3. Before concluding the article, the limitations and potential directions of proposed reviews are reported in Section 4.

## 2. Review Design

### 2.1. Finding Relevant Literature

**Figure 1:**
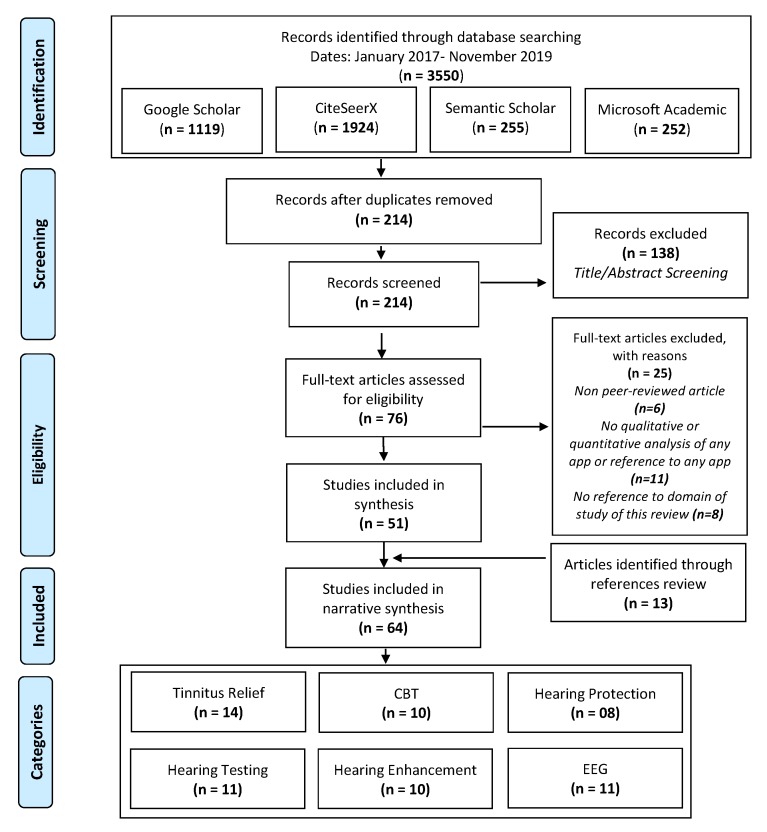
Prisma workflow for systematic review.

The workflow diagram for the systematic identification of scientific literature is illustrated in Figure 1. The sources (Google Scholar, CiteSeerX, Semantic Scholar, and Microsoft Academic) were queried to find relevant literature from 2017 and onwards. The keywords used to perform searches are (tinnitus OR hearing) AND (smartphone OR mobile) AND (Apps OR systems). Two separate cycles of searches were performed on different dates: (1) 15May2019, and (2) 15November2019. Finally, the results were fused together, duplicates removed and prepared for further screening.

A total of n=214 records were considered for screening in the identification phase (Figure 1). In a further step, a screening was performed on the titles and abstracts of these selected records for eligibility, which resulted in the feasibility of n=76 records for further evaluation. The full texts of the selected 76 records were then assessed for further suitability, resulting in a rejection of further 25 records due to several reasons: 6 out of the 25 records were not subjected to a peer-review process. 11 records did not perform any qualitative or quantitative analysis of the respective app or did not reference any app. 8 records did not show any meaningful overlap with the content, aim and scope of this review. Finally, the review selection process resulted in the inclusion of 51 records, whereas 13 articles were additionally added through a review of references. Finally, the total number of included records was, therefore, n=64. The identified literature has been subsequently categorized with the help of tinnitus experts into six topics: 1) tinnitus relief (n=14), 2) CBT (n=10), 3) hearing protection (n=08), 4) hearing testing (n=11), 5) hearing enhancement (n=10), and 6) smartphone-based mobile EEG systems (n=11).

### 2.2. Finding Relevant Apps

**Figure 2:**
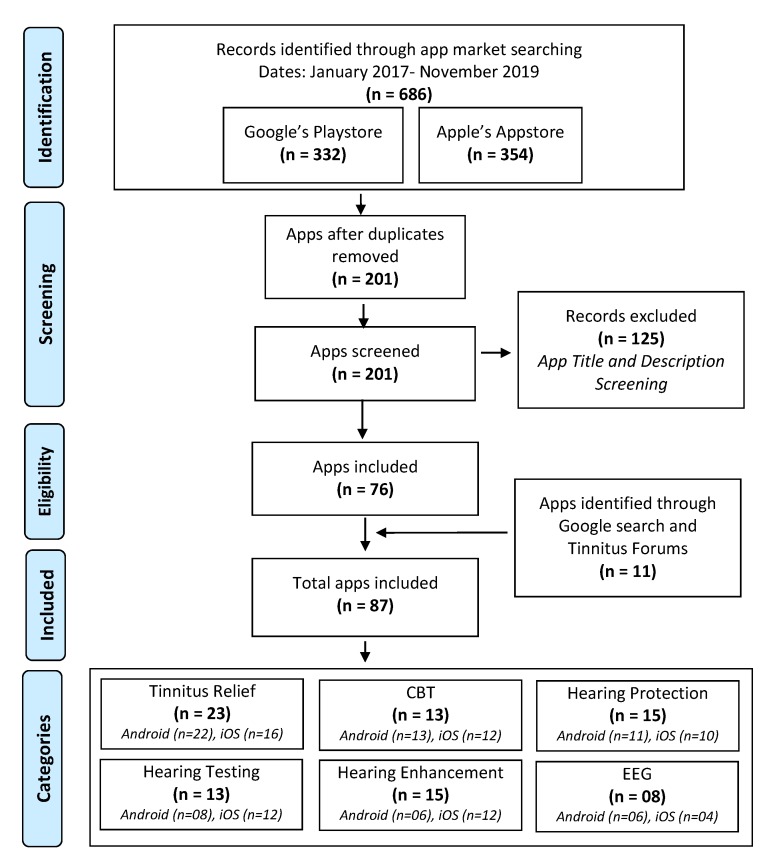
Prisma workflow for systematic review—apps.

The overall process of systematically identifying relevant apps is illustrated in Figure 2. The two aforementioned app stores (Google’s Play Store and Apple’s App Store) were searched to cover both major mobile platforms (i.e., Android and iOS). Due to device-specific limitations of apps from different app stores, we did not consider app stores like Amazon Appstore, Sony Apps, Samsung Galaxy Apps, Huawei App store, and LG SmartWorld into our app search workflow. Furthermore, third-party app providers like Aptoide or F-Droid were not considered as reliable sources due to (general) security issues and their reliance on rooted devices. Rooting is the process of acquiring full system access or administrative control of mobile devices. This process is highly discouraged by device manufacturers and app developers as it introduces security vulnerabilities [36].

Consequently, the combination of keywords tinnitus, hearing protection, hearing enhancement, noise exposure, CBT, self-help were used for the search procedure. The search yielded a total sum of 686 apps on both app markets, where 332 apps were found on Google’s Play Store, and 354 apps were found on Apple’s App Store. 201 apps were filtered out after removing duplicates appearing in both app stores in the identification phase. The 201 apps were then screened based on the title and app description, resulting in the feasibility of n=76 apps. Secondarily, using the same keywords (appended by the keyword ‘app’), Google searches were performed to find any missing or additional app (two runs in May 2019 and November 2019). The Google search yielded multiple web pages, blog posts and tinnitus forums. The content of all three were investigated manually to identify potential relevance and relevant apps, resulting in the identification of 11 additional apps. Finally, a total of 87 apps were included in this review. The distribution of the identified apps in aforementioned categories is as: (1) Tinnitus Relief (n=23), (2) CBT (n=13), (3) Hearing Protection (n=15), (4) Hearing Testing (n=13), (5) Hearing Enhancement (n=15), and (6) Smartphone-based Mobile EEG Systems (n=08).

### 2.3. Rationale Behind App Categorization

In addition to the apps related to tinnitus relief, we report on apps that provide CBT or self-help, helpful for hearing loss as it is one of the major causal risk factors [37,38], and smartphone-based mobile EEG systems. The rationale behind the categorization of apps into six topics identified through literature screening and surveying the apps stores are further detailed below:

**Tinnitus** **Relief** Different treatment modalities for the management of tinnitus symptoms exist, for instance, Tinnitus Retaining Therapy (TRT), Tinnitus Masking (TM), conventional drug delivery, and even brain stimulation—among them, TRT, TM using sound generators, and CBT as counseling are standard treatment procedures [1]. Most of the tinnitus relief apps that are generally published on app markets offer tinnitus masking, or sound therapies using different sound techniques like acoustic neuromodulation, notched sound, or amplitude modulation. Smartphones are capable of delivering acoustic and sound therapy reliably and accurately [39].
**CBT** Although, the acoustic characteristics of tinnitus, particularly the subjective loudness of tinnitus is minimally affected by CBT [40], CBT has been pivotal for the treatment of tinnitus [41]. Since CBT and self-help therapies have been useful in dealing with stress and anxiety associated with tinnitus [42], we deem it important to be included in this review.
**Hearing** **protection** Tinnitus is reported to be accompanied by hearing loss in more than 80% of cases [29]. Augmentation of tinnitus symptoms with increased noise exposures [43], and hearing loss being prevalent causal risk factor for tinnitus [44], it can be argued that hearing protection can lead to reduced odds for developing tinnitus, as well as support tinnitus patients in managing their symptoms.
**Hearing** **testing** Since hearing loss is a commonly occurring phenomenon with tinnitus, we argue that apps for hearing testing are certainly linked to processes of tinnitus matching (for example, sensation levels for staircase procedures). Therefore, smartphone-based tinnitus matching may be a relevant feature for current or future sound therapies. Furthermore, in patients where the tinnitus is caused by hearing damage, using hearing aids (or even cochlear implants) can help to reduce tinnitus symptoms [45]. Therefore, we believe that it makes sense for the tinnitus patients to test their hearing and thus hearing testing apps are relevant for this review.
**Hearing** **enhancement** Tinnitus and hearing loss has been reported to directly influence the quality of life of patients [44]. Apps for hearing enhancement could be useful to counteract tinnitus-related impairments of hearing functions in daily life like speech in noisy environments or cocktail-party situations, and directional hearing. Therefore, we consider our addition of hearing enhancement as rather straight forward.
**Smartphone-based** **EEG** Despite the fact that tinnitus is traditionally considered only a problem of the inner ear, research using brain imaging has shown that the complexity of tinnitus goes beyond the auditory cortex into non-auditory brain areas [43,46]. Additionally, EEG allows the investigation of resting-state activity of the brain [43], and is widespread in tinnitus research [47,48,49]. With the growing interest in the development and significant technological advancements in mobile-based EEG systems and due to the fact of recent developments, it is possible to record EEG outside of a laboratory setting. Further, EEG is one approach of few to record the changes in brain activity, which corresponds to the perception of tinnitus [49]. That is why we considered smartphone-based electroencephalography (EEG) to be pertinent and included it in this review.

## 3. Results and Discussion

### 3.1. Tinnitus Relief Using Smartphones

A significant portion of scientific literature within tinnitus research reports on different applications of smartphone apps and mobile crowdsensing, ranging from data collection to mitigating tinnitus symptoms via therapeutic interventions and supporting clinicians in better understanding the tinnitus. These applications are specifically designed to assist patients, clinicians, and researchers alike.

From the perspective of patients, these apps aim to assist patients in masking, controlling, mitigating, or managing tinnitus symptoms by means of providing smartphone-delivered CBT, tinnitus or sound therapy, or keeping track of individual tinnitus perception using standard questionnaires. For instance, the smartphone app TrackYourTinnitus (TYT) [50] systematically records fluctuations of tinnitus symptoms over time from patients using Mini-Tinnitus Questionnaire (Mini-TQ) [51] and Visual Analogue Scale (VAS) [52]. Both Mini-TQ and VAS are employed by TYT app to acquire tinnitus-related data such as tinnitus presence, stress, loudness, and tinnitus-related distress. The aggregated data from the app provides information about a patient’s tinnitus variability over time, thus, enabling patients to easily identify critical circumstances causing fluctuations in their individual tinnitus perception [20]. This helps patients to not only have necessary knowledge and information regarding their tinnitus but also assists them to demystify the tinnitus symptoms and establish control over tinnitus. Similarly, Ref. [53] delve into the development of an app, based on the progressive tinnitus management to support patients in learning and using coping skills for tinnitus management. Ref. [54], in turn, outlines a self-management tinnitus app that combines audiometric examinations and administration of questionnaires, namely the Pittsburgh Sleep Quality Index, PSQI, [55]; Khalfa Hyperacusis Questionnaire [56]; Tinnitus Handicap Inventory (THI) [57]. The authors employ and present an external device for audiometry testing and argue that the app supports patients with their diagnostic procedures.

From the perspective of clinicians and researchers, these apps enable and support a better understanding of different aspects of tinnitus. Among many, some of the most important ones are to better understand and identify tinnitus severity and tinnitus behavior in different patients and to better understand the tinnitus heterogeneity in general. For instance, the data collected from the TrackYourTinnitus app can be used to establish a connection between tinnitus and daily routine or activities [58]. Additionally, the same data can be analyzed to shape recruiting strategies for larger tinnitus-related studies [59], and to better understand tinnitus variability and tinnitus–stress associations [60]. Similarly, the similarities and differences in ecological momentary assessment data, collected from the TrackYourTinnitus app after a long time can also help physicians to understand the evolution of tinnitus patients [61].

In terms of tinnitus-related therapies to control tinnitus symptoms, Ref. [62] highlight the use of sound-related therapy. Ref. [63] aims to assess and review smartphone-app–supported therapies for tinnitus, which is argued to be useful, additionally to multimodal tinnitus therapies. In another study, Ref. [64] employ the use of a smartphone app to deliver notched music to tinnitus patients. It is argued in the study that the overall THI scores (emotional score of THI in particular) of tinnitus patients improved.

A comprehensive list of apps that assist patients in providing tinnitus-related relief is shown in Table 1, with their respective properties. Among the app properties, we identify that the users-property will provide the readers with coverage of the apps’ usage. Please note that in the case of the iOS platform, the number of users is not publicly provided by the app store. Rating will highlight the impact of the app according to the app store’s rating system, update gives the last seen update on the corresponding store. Pricing will give insights into the economical aspects of apps. The app price is given in Euros, whereas for free apps, further categories exist: Free (I) corresponds to apps that are available free with purchases inside the app (in-app purchases), and Free (A) corresponds to apps that are supported by advertisements (ad supported). Please note that free apps can also come with both combined, denoted as Free (I,A). The platform property is necessary as it explores the platform-specific user base and their behavior towards the app. For instance, a platform-specific data-analysis-based comparison of TrackYourTinnitus is given in [65], in which the authors aim to highlight the differences between iOS and Android users to better understand their use of mobile apps within tinnitus context. The difference in platform-specific user behavior to an app is also evident in Table 1 when comparing the ratings of TrackYourTinnitus app.

Further, note two important aspects for the app list shown in Table 1: the list refers to the selected search criteria for the PRISMA guidelines, it neither claims to be complete nor can it reveal which apps are more evidence-based as others. Although new rating systems like the Mobile App Rating Scale (MARS) aim to establish standards in this context, their widespread use is still not given. Therefore, systematic reviews like presented in the work at hand are currently a key factor for beneficial insights on mobile health apps.

### 3.2. Smartphone-Based CBT

In addition to sound-related therapies, Cognitive Behavioural Therapy (CBT) has been pivotal for the treatment of tinnitus [41]. Although it is argued that CBT has no effect on the acoustic characteristics of tinnitus, such as subjective loudness of tinnitus [40,42]. However, it has proven to be effective in improving the overall quality of life of tinnitus patients and reducing symptoms of tinnitus-related psychological comorbidities, such as depression and anxiety [42,66]. Besides CBT being administered face to face with a CBT clinician, it can also be administered via the internet in the form of self-help treatment for tinnitus [67]. Evidence from the literature suggests that internet-delivered self-help tinnitus treatment shows positive results and is an effective treatment modality [68,69]. Consequently, the smartphone app markets have a variety of apps that are specifically designed for CBT for tinnitus, such as (Beltone Tinnitus Calmer, Diapason for Tinnitus, ResoundRelief). Moreover, general CBT self-help apps can be adapted to accommodate tinnitus-related relief, for instance, to manage and control depression and anxiety, two of the most common and prevalent comorbidities accompanied by tinnitus [70,71].

Our literature search did not yield any specific clinical validation studies of CBT apps directly applied to tinnitus. However, in one study given in [72], the effectiveness of CBT in tinnitus has been discussed and has shown positive results. The participants of the study reported reduced TFI scores after receiving mindfulness-based CBT therapy. The study further discusses the beneficial effects of mindfulness-based CBT in relation to distress associated with tinnitus. Although the study does not show the usage of any specific app, mindfulness-based CBT is delivered by most of the apps listed in Table 2.

### 3.3. Smartphone-Based Hearing Protection

In addition to apps that support patients to manage tinnitus, the embedded microphone of a smartphone can be used as a valid device to detect sound exposures. Herein, many smartphone developers have contributed to the app market; i.e., with apps ranging from ones that record, visualize, and report sound exposures to ones that help to find a quiet place. We have identified such apps and summarized them in Table 3. For this review, we do not list apps that are solely used for the purpose of performing sound recordings, but the focus is to have additional functionality to monitoring sound levels. For instance, an app that records sound levels and propagates the recordings to a database, resulting in a crowdsourcing-based noise level detection in different places or an app that detects sound exposure and notifies the user of dangerous sound levels.

Furthermore, extensive literature development on hearing protection using smartphone technology exists. In terms of education to preserve hearing or perform hearing protection interventions, in [73], the authors report on the use of technology-based interventions to improve hearing protection in adolescent farmworkers. The study compares the use of technology (computer- and smartphone-based) vs face-to-face training modalities for hearing conservation and protection. The six-week study yielded statistically non-significant changes in the user’s attitude, behavior, and knowledge in terms of hearing conservation education in three groups (computer, smartphone, face-to-face). The results from pre- and post-intervention survey of 70 participants (only 50 participants responded to post-intervention survey) established that the hearing conservation knowledge of participants improved for all three groups, but fails to discuss if technology-based interventions were better than that of face-to-face. Another article presented by [74] adopts a military hearing conservation program namely, military hearing conservation programs (MHCPs) into a smartphone app. In addition to listing and reviewing existing smartphone apps for hearing protection, the article discusses the technical details of the development of the Warfighter’s Hearing Health Instructional (WHHIP) app. The article lapses in critically evaluating the developed app both in terms of technical as well as presents no data or results on the effectiveness of the developed app. Although, smartphone-delivered hearing conservation training and educational apps on hearing protection can be a cost-effective alternative mode to face-to-face training, particularly in a remote location setting, the effectiveness of involving such technological tools is still not established [75].

The following articles feature the technologies and subsequent improvements for hearing-loss protection: [76] points out the technological advancements for hearing loss prevention. Herein, in addition to listing some smartphone-based apps, the authors talk about the AI-based solutions in hearables (such as smart headphones or earphones). The article also discusses the issues of work-related noise and how to tackle it by employing technological tools to prevent hearing loss. Similarly, Ref. [77] explores the relation between noise-induced hearing loss and occupational noise exposure, the study investigates the use of smartphones to measure sound exposure or noise in occupational settings. In addition to highlighting some smartphone apps, the author conducts multiple experiments using different combinations of microphones, occupational settings, and sound measuring apps. In the opinion of the author, smartphones tend to overestimate noise exposure by significantly lower margins making mobile-based audiometry an accurate alternative for noise-level detection. Ref. [78] compare a Sound Level Meter app developed by the National Institute for Occupational Safety and Health (NIOSH) in measuring industrial/mining sound levels as opposed to controlled laboratory environments. The authors conclude that the use of apps in industrial/mining settings as being useful with recommendations for additional validations. In their observational study, [79] primarily determined if the smartphone app (SoundMeter Pro app) could be useful for detecting dangerous sound-levels based on NIOSH guidelines. Secondarily, the study evaluates noise exposure measurement in exercise spin classes, concluding that the exercise classes generate noise levels that can induce hearing loss and that the use of mobile-based audiometry allows real-time monitoring of noise exposures. Despite the fact that smartphone-based noise exposure detection offers viable and accurate technique, and some smartphone apps for noise exposure detection have been validated in literature [80], hearing healthcare professionals have to be involved in the process to ensure that these technological advancements are properly employed to avoid hearing loss.

The literature on applicability and validation of hearing protection particularly for tinnitus patients is almost non-existent. However, it is established that exposure of tinnitus patients to dangerous levels of sound usually results in increased tinnitus symptoms, causing increased tinnitus-related distress and anxiety. The commonly occurring hearing loss with tinnitus can further attribute to increasing this annoyance and distress [10]. The aforementioned literature pertaining to mobile-based audiometry and sound-level monitoring suggests that smartphone apps can enable users to monitor sound levels with minimum error and take necessary countermeasures to prevent dangerous noise exposures. Additionally, the smartphone apps highlighted in this subsection can enable tinnitus patients in attaining necessary knowledge about managing their hearing loss with the help of hearing conservation programs. The knowledge of current sound environment using these smartphone apps can also help tinnitus patients in preserving the remaining hearing, and manage tinnitus symptoms.

### 3.4. Hearing Testing Using Smartphones

Testing hearing or audiometry may be achieved via smartphone apps. Given that the audiometry is properly performed, smartphone-based solutions may prove to be useful in resource-limited settings. An ample amount of hearing testing apps exist in the smartphone market places. A list of apps that provide hearing testing are given in Table 4. These apps can range from testing hearing on an individual level to comparing testing capabilities with others (for example, family members or friends). Furthermore, they can be used to test hearing on different frequencies, as well as to test hearing in noisy environments.

A considerable amount of peer-reviewed literature has reported on testing hearing with the help of smartphones. Some of this literature probes the applicability of mobile hearing testing in young adults or children. The paper presented by [81] is a case in point, where the authors outline a smartphone-based hearing screening method. The authors explore and compare their proposed smartphone-based hearing screening app (Ear Scale app) with Pure-tone Screening (PTS) in a sound-treated booth to test the hearing of school-age children. The detailed evaluations presented in the paper suggest that the Ear Scale app was able to accurately measure hearing loss (moderate to worse) in school children and that the hearing screening performed using the app showed positive consistency with PTS. Similarly, Ref. [82] propose an Android-based app to screen the hearing of pre-school-aged children. The proposed app showed children with different pictures to choose, based on the word they hear. The predefined set of words, in turn, is describing the pictures heard by children on different sound levels. The outcomes of the proposed methods were compared with conventional audiometric methods as well as alternative smartphone-based audiometric apps. The final developed mobile device-based screening system (PASS Speech Audiometry Version 2) suggests that smartphone-based audiometry can be easily adopted to prevent and manage hearing loss. Furthermore, smartphone-based hearing testing can also be a viable and cost-effective solution for accurately conducting audiometry in community-based early childhood development centers, particularly in poor communities [83]. To add to this, smartphone-based audiometry can also be used to estimate pure-tone thresholds [84].

Moreover, evidence of use of smartphone-based hearing testing in clinical settings has also been reported. An evaluation study presented by [85] tests the performance of the hearScreen™ app at two primary health care clinics. The sensitivity and specificity analyses of the hearScreen™ app suggests that the smartphone-based hearing testing within clinical settings is n adequate tools. However, the role of audiologists remain significant while interpreting data from these apps to make clinical decisions [86]. Within an Infectious Disease (ID) clinic setting, another article evaluated the hearTest™ app as a clinical utility and determined that smartphone-based hearing screening can be a valid baseline tool [87]. The hearTest™ app is further validated using calibrated supra-aural headphones and inexpensive smartphones in [88]. In conclusion, the hearTest™ app can be used to determine valid air-conduction hearing thresholds. In another validation study [89], the hearTest™ app was validated for extended high frequency hearing thresholds, determining that calibrated headphones while used in combination with the app provide accurate and reliable results.

Evidently, a critical aspect of smartphone-based hearing testing is the use of headphones or earphones. Usually, prior to conducting a hearing test, users are required to calibrate either device-provided or model-specific headphones (bundled), or any other non-bundled headphones. The calibration is performed by a normal-hearing person in order to determine a reference sound level for comparison. A comparison of pure-tone audiometry hearing thresholds with the hearing threshold measured via smartphone is done in [90]. In this scenario, the comparison is done on a smartphone calibrated using bundled headphones and biologically determined reference sound levels. The authors report that hearing testing on smartphones with bundled headphones are highly compatible with pure-tone audiometry. A detailed evaluation of four different headphone models that are used with mobile-based hearing testing apps concluded that mobile-based hearing testing produces audiologists-quality data when coupled with suitable headphones [91].

Although hearing loss is attributed to increasing tinnitus-related annoyance, the levels of hearing loss in different tinnitus patients vary, particularly for patients whose tinnitus is caused by hearing damage [1]. The smartphone apps reported in this subsection can help these tinnitus patients in identifying their specific hearing loss to apply the best possible therapy (for instance, sound therapy) and deplete tinnitus-related symptoms. Smartphone-based hearing testing apps have been reported in peer-reviewed literature discussing and evaluating their applicability in different scenarios (for example, clinical settings, remote urban locations), however, the validity of these apps is still under argument [35]. Despite the fact that hearing loss is commonly occurring phenomenon with tinnitus and different other disorders like hyperacusis and Meniere’s disease, the literature on the validation of smartphone-based hearing testing apps is undeniably non-existent. Therefore, in our opinion, a detailed validation of smartphone-based hearing testing apps is indispensable, specifically for tinnitus patients.

### 3.5. Smartphones-Based Hearing Enhancement

The embedded microphone in the smartphone combined with headphones can sustain hearing enhancement in patients who are suffering from hearing loss. In this subsection, we discuss the literature and smartphone apps pertaining to hearing enhancement using smartphones. The list of apps identified during the review process are listed in Table 5. It is critical to note here that for this review, we do not cite literature that reports on the use of hearing aids, even if they are optimized or tailored to be used with smartphone apps. For insights into hearing aids, we encourage readers to have a look at [92]. In Table 5, we specify smartphone apps based on two major aspects, namely the simple boosting of the audio signal or specific frequencies as well as the filtering out of distracting noise. Recall that most smartphone apps provide accurate and reliable results while used in combination with bundled headphones, therefore, we have limited the scope of this review to smartphone or headphone combinations for hearing enhancement, particularly because most commercially available smartphones come with bundled headphones.

The application of smartphones targeting hearing enhancement can range from smartphone-based games, up to auditory training programs, all the way to implementing a digital hearing aid with the mobile device. An example implementation of smartphones as digital aid is discussed by [93]. The authors employ an audio-signal processing technique to develop a smartphone app enabling the device to be used as hearing aid. Even though the final developed app has limited real-life evaluation and validation, the results suggest that the app has significantly low latency and therefore is a viable solution for face-to-face conversation. Ref. [94] detail a smartphone app based on audio signal processing, where three important and generally used modules in digital hearing aids (namely, voice activity detection, noise reduction, and compression) are implemented. Although the article discusses the technical details of implementing the digital hearing aid modules, the final developed systems are not subjected to any evaluation and therefore no data or results on the accuracy of the implemented algorithms are discussed.

Noise reduction can be a critical aspect in improving overall hearing enhancement experience. Generally, the noise reduction is carried out using an algorithm (for example, Binaural noise reduction algorithm [95]), or using an unsupervised or supervised classifier [96]. Ref. [97] proposes an app that is capable of achieving real-time noise reduction of speech signals, particularly in noisy sound environments. The app relies on the Wiener Noise Reduction algorithm and the authors report positive effects of noise reduction via the proposed app based on objective and subjective evaluation. Analogous to this, and implementation of a smartphone app to classify noise by unsupervised classifier is detailed by [98]. The aforementioned modules used in digital hearing aid implementations (namely, voice activity detection, noise reduction, and compression) can be combined with this noise-classification app to further improve the audio signal processing pipeline.

Furthermore, in addition to the apps mentioned in Table 5, smartphone apps also exist as auditory training programs to assist patients with hearing loss [99]. Alternatively, smartphones can be assistive in improving auditory memory skills. Ref. [100] proposes the process of implementing a smartphone app that enables improvement of auditory memory skills, particularly in children with hearing loss. The authors report that six different smartphone apps were developed, for all of which the usability tests were conducted, and the results suggest that the developed apps were suitable in improving auditory memory skills for children efficiently.

Tinnitus is known to impact the quality of life of tinnitus patients. Although, the number of patients who experience a drastic decrease in their life quality is some-what low [1], almost all patients suffering from tinnitus are affected by the annoyance caused by tinnitus in different life situations [101]. For instance, the continuous perception of phantom sound in the ear, and the usually accompanying hearing loss can negatively affect a person in a social interaction scenario or in a working environment, resulting in reduced socialization [102]. The smartphone-based hearing enhancement apps can certainly assist patients in improving and managing such situations and a cost-effective alternative to clinically validated cochlear implants or hearing aids (both clinically validated techniques to improve the quality of life of tinnitus patients [103,104]). Further, as these smartphone apps can be used as a digital hearing aid, or alternatively by achieving hearing enhancement using sound amplification techniques. However, it should be noted that our literature search and review didn’t reveal any study that validated the use of hearing protection apps in the tinnitus context. There is a dire need for studies to validate and compare smartphone-based hearing enhancement techniques with cochlear implants and hearing aids. Additionally, the smartphone apps need to be validated in terms of applicability and their use in tinnitus, as enhancing hearing or sound amplification—despite being beneficial—can inadvertently cause further damage (sound is a major factor in causing tinnitus-related discomfort).

### 3.6. EEG Systems and Smartphones

Tinnitus is perceived as ringing or buzzing located in one, both or between the ears. However, the underlying effects of generation and manifestation pertain to the complete auditory pathway and are not completely identified until today. Despite the unknown, a general conception of tinnitus is the idea that the central nervous system is involved, and that tinnitus has its own neural correlates [105]. Among others, oscillatory alterations have been identified in EEG studies [49]. Despite contradictory results, there have been recent developments suggesting Neurofeedback (NFB) as a suitable treatment alternative [106].

NFB is a non-invasive method, generally based on EEG recordings, which are analyzed in real-time, visualizing certain aspects of brain activity (for example, frequency band power) as a positive or negative feedback that enables users or participants to voluntarily control their brain activity patterns. While its mechanisms of action are still under scrutiny [107], NFB seems to be a viable candidate for interventions in tinnitus and other chronic diseases. Available NFB applications in the open market are primarily offered for concentration and meditation exercises and less for the medical field. Yet, we do not want to leave this point unconsidered, and thus we take a step back to look at available EEG apps in the hope that future NFB apps, with scientific claims, could be developed on this basis.

To assess the oscillatory fingerprint of diseases like tinnitus, EEG recordings were performed in a laboratory setting so far. Recently, researchers are increasingly attempting to transfer EEG recordings from this static to novel environment like the private setting (for example, at home). This so-called “mobile EEG” is an increasingly active area including the development of brain-computer interfaces (BCI) for a vast range of non-medical applications, such as gaming [108], meditation, sport activities [109], and personal health, for instance ‘brain training’ like neurofeedback [110,111]. In this section, we will identify the apps being offered in the context of mobile EEG recordings (see Table 6).

Over the past few years, research showed that the signals acquired via wireless mobile EEG headset devices are similar to those obtained with standard EEG laboratory equipment and traditional rating scales or diagnostic assessments [112,113]. Due to the increasing interest in mobile EEG applications, our search revealed several EEG apps, but not many were designed for research or medical applications. From the latter group, most apps require, or are designed for, specific kinds of EEG headset devices. These devices run on wet or dry electrodes and typically include impedance differential inputs, an electrode ground, as well as other principal features required for EEG recordings. For a comprehensive review, readers can refer to an existing coverage [114]. Concurrently, the apps’ software either stores the raw data with a download option (EEG Analyzer, Muse Monitor, BrainLog) or supports ’online data processing’-tasks, such as acquisition, recording, or breaking down the EEG signal by different scalp potentials in either frequency bands (eegID) or in “cognitive and emotional metrics” as it is advertised by other applications, for instance, MyEmotiv. An important advantage of some of the apps is the open-source software code, which can be downloaded and adapted to personal needs (for example, EEG 101).

Mobile EEG systems and their applications offer great potential, not only, but also for the field of tinnitus. However, in the context of medical and health-related services, a quality criterion such as the CE mark, has not yet been defined in mobile applications. It, therefore, seems important that, in addition to an authority overseeing the application software, highly qualified players in the health-care system should be involved in the creation of such apps.

## 4. Limitations, Future Work and Conclusions

Relevant literature search—we are aware that keyword-based search can have limited coverage, because there may be relevant documents not matching the chosen keywords. Different search results obtained by the same query terms applied on different data sources gave some hints that query terms could be further optimized. We attempted to improve this by isolating keywords that caused reduced recall, however, we believe that it can be further improved. Similarly, most of these searches were performed using the keyword ‘tinnitus’, this, on one hand, increases the precision to find tinnitus-related studies, however, on the other, it limits the identification of studies reporting on closely related subject areas to tinnitus. For instance, adding the ‘hearing loss’ keyword in the search criteria could benefit from increasing the recall. Furthermore, we thoroughly ensured the selection of relevant literature based on primarily investigating the abstract and introduction for relevance, and secondarily based on the content of the paper. Again, we are aware that this process is subjective and can probably be further improved.

Identifying relevant apps—another limitation of our proposed work constitutes the restricted search of relevant apps in only two app stores, namely Google’s Play Store and Apple’s App Store. Even though we justify this restriction, there is a slight chance that our work could benefit by exploring other app stores, like Amazon’s and Samsung’s app stores. Although unlikely, 3rd party independent app stores like Aptoide or F-Droid may contain previously unseen apps. During our searches, we identified apps which were relevant for this review and were part of Google’s Play Store or Apple’s App Store at one point of time, however, they were removed from respective app stores due to policy conformation issues. Usually, removal of an app from these app stores is properly justified, however, these restrictions can sometimes be inconsistent. Furthermore, to include an app in this review, we inspected the app description and a selection of a few top-rated comments from users. This approach is subjective and highly relies on the knowledge of the inspector about the domain and can be further improved by collecting opinions from domain experts as opposed to general users.

For prospective work, we primarily aim to extend our work by reviewing internet- and computer-based behavioral therapies applied directly in the context of tinnitus research. Herein, an additional focus would be to include studies that report on the use of auxiliary and peripheral sensors in assisting therapeutical solutions. For instance, the use of smartwatches or wristbands to acquire physiological attributes of patients suffering from tinnitus could be additionally included. Secondarily, as future work, we plan to extend our work by addressing psychological conditions that are closely related to tinnitus. In view of this, the objectives of the study would be to identify smartphone-based solutions, as well as internet- and computer-based therapeutical solutions offered for the said complications. Furthermore, the study will report on the importance of highlighted therapies and opinions of smartphone-based solutions from the perspective of patients.

In conclusion, the review presented in this paper highlights the impact of mobile applications and mobile crowdsensing platforms, specifically within the context of tinnitus research. We identified and investigated a wide array of heterogeneous apps heavily invested in supporting and controlling tinnitus symptoms, understanding tinnitus, and monitoring patients suffering from tinnitus. Primarily, we highlighted the available mobile apps that could be beneficial for a patient in mitigating or masking annoying phantom sounds. We further explored different smartphone-delivered CBT therapies that can be adopted by tinnitus patients to manage stress and anxiety accompanied by tinnitus. As significant and continuous exposures to dangerous levels of sound are critical aspects in increasing tinnitus symptoms or causing them, secondarily, we scrutinized mobile apps that are helpful in monitoring noise levels and noise exposure, including apps that notify the user of dangerous exposure. Moreover, we inspected apps that are useful in testing hearing. This is beneficial for patients suffering from tinnitus by allowing them to monitor the hearing loss commonly occurring with tinnitus. Furthermore, we explored and presented apps that provide assistance to patients suffering from hearing impairment. For all categories, we not only provided a list of available apps but also reviewed relevant literature documenting the usage and role of those apps.

## Figures and Tables

**Table 1:** Apps providing tinnitus-related relief (Free = royalty free, Free(I) = in-app purchases, Free(A) = ad-supported, Free(I,A) = both) *Apps reported in literature.

App Name	Description	Platform	Users	Rating	Update	Pricing
**H& T Sound Therapy**	Noise Player (pink noise, white noise or brown noise) for masking tinnitus	Android	10K+	4.3/5.0	Oct-19	Free
Kalmeda mynoise*	Offers medically-based, individual tinnitus therapy	Android	1000+	3.0/5.0	Jul-19	Free(I)
		iOS	-	3.6/5.0	Jul-19	Free(I)
myNoise*	Controlling tinnitus via combination of different sounds and noises	Android	100K+	4.4/5.0	Mar-18	Free(I)
		iOS	-	4.6/5.0	Apr-19	Free(I)
Oticon Tinnitus Sound*	Offers different sound types to control tinnitus	Android	100K+	2.0/5.0	Feb-19	Free
		iOS	-	2.9/5.0	Feb-19	Free
Relax Melodies*	Sleep assisting app that combines sounds and melodies	Android	10M+	4.6/5.0	May-19	Free(I, A)
		iOS	-	4.8/5.0	May-19	Free(I)
Relax Noise 3*	Masking tinnitus by using red, white, or pink noise	Android	100K+	4.2/5.0	Mar-15	Free
SimplyNoise*	Controlling and managing stress and tinnitus using white, and brown noises	Android	50K+	3.7/5.0	Jun-12	Free
		iOS	-	4.4/5.0	May-18	Free(I)
Starkey Relax*	Tinnitus masking, self-management, and education app	Android	10K+	4.3/5.0	Oct-17	Free
		iOS	-	3.9/5.0	Oct-17	Free
StopTinnitus*	Masking tinnitus using customised tones	Android	100+	2.7/5.0	Jan-15	7.95
		iOS	-	1.3/5.0	Jan-15	8.03
Tinnitracks*	Controlling and managing tinnitus by filtering out music for sound therapy	Android	10K+	3.8/5.0	Apr-19	Free(I)
		iOS	-	3.6/5.0	Feb-19	Free(I)
Tinnitus Balance App*	Controlling annoying tinnitus using customised sounds or music	Android	50K+	3.7/5.0	Mar-16	Free
		iOS	-	2.3/5.0	Mar-19	Free
Tinnitus Help*	Tinnitus masking using natural sounds or music	Android	500+	3.0/5.0	Nov-15	9.90
		iOS	-	4.4/5.0	Jan-19	17.99
**Tinnitus Notch**	Provided custom tailored notch therapy for tinnitus relief	Android	1000+	2.7/5.0	Sep-16	Free(I)
**Tinnitus Peace**	Offers melodies to match the frequency of tinnitus to reduce its effects	Android	5K+	3.8/5.0	Nov-15	Free
**TinnitusPlay**	Tinnitus masking using different sound techniques	iOS	-	4.2/5.0	Dec-19	Free
Tinnitus Relief*	Controlling tinnitus using information on different relaxation exercises	Android	1000+	4.4/5.0	Dec-13	2.99
**Tinnitus Sound Therapy**	Sound/Acoustic therapy for masking tinnitus	Android	10K+	3.9/5.0	Jun-19	Free
Tinnitus Therapy (Lite)*	Avoiding tinnitus with sound masking and therapy	Android	500+	3.6/5.0	Feb-19	6.49
		iOS	-	5.0/5.0	Mar-19	5.36
Tonal Tinnitus Therapy*	Helps to mitigate symptoms of tonal tinnitus based on acoustic neuromodulation	Android	10K+	4.0/5.0	Jul-18	Free(I)
Track Your Tinnitus*	Managing tinnitus by tracking tinnitus patterns in daily activity	Android	1000+	2.1/5.0	Oct-18	Free
		iOS	-	5.0/5.0	Jun-17	Free
Whist*	Controlling tinnitus using sounds with adjusted volume, pitch etc.	Android	1000+	4.2/5.0	Mar-17	2.18
		iOS	-	3.7/5.0	Jan-19	1.78
White Noise (Lite)*	Masking and Controlling tinnitus using environmental sounds	Android	5K+	4.6/5.0	Sep-18	3.19
		iOS	-	4.8/5.0	Apr-19	2.67
Widex Zen*	Avoiding tinnitus using relaxing zen sounds, and exercises to manage tinnitus	Android	10K+	3.8/5.0	May-17	Free
		iOS	-	5.0/5.0	Nov-17	Free

**Table 2:** Apps providing CBT (Free = royalty free, Free(I) = in-app purchases, Free(A) = ad-supported, Free(I,A) = both). *Apps reported in literature.

App Name	Description	Platform	Users	Rating	Update	Pricing
Beltone Tinnitus Calmer*	Combination of relaxation exercise and sound therapy to avoid tinnitus	Android	1000+	4.7/5.0	Sep-19	Free(I)
		iOS	-	5.0/5.0	Sep-19	Free(I)
**CBT Companion**	Employs visual tools to learn and practice CBT techniques	Android	50K+	4.6/5.0	Feb-19	Free(I)
		iOS	-	4.7/5.0	Feb-19	Free
Diapason for tinnitus*	Game-based digital therapeutic providing app for tinnitus relief	Android	5K+	3.1/5.0	May-19	Free(I)
		iOS	-	-	May-19	Free(I)
MindShift CBT*	CBT tools to manage and control anxiety	Android	100K+	3.9/5.0	Oct-19	Free
		iOS	-	4.2/5.0	Oct-19	Free
**Moodfit—Stress and Anxiety**	Stress and Anxiety management and tracking, and offers CBT exercises	Android	5K+	4.4/5.0	Aug-19	Free
**Quirk CBT**	Self-help CBT companion based on ‘three column technique’	Android	10K+	3.6/5.0	Jul-19	Free(I)
		iOS	-	4.7/5.0	Sep-19	Free(I)
ReSound Relief*	Avoiding tinnitus using combination of sound therapy and relaxation exercise	Android	100K+	4.5/5.0	Feb-19	Free(I)
		iOS	-	4.7/5.0	Jan-19	Free(I)
**Sanvello—Stress and Anxiety Help**	Audio and Video CBT exercises, Anxiety tracking and management	Android	1M+	4.6/5.0	Feb-19	Free(I)
		iOS	-	4.8/5.0	Nov-19	Free(I)
**Stress and Anxiety Companion**	CBT based visual exercises to manage stress and anxiety	Android	10K+	4.2/5.0	Jul-19	Free(I)
		iOS	-	4.6/5.0	Jun-19	Free(I)
**What’s Up? A Mental Health App**	Offers CBT and ACT methods to manage stress, anxiety as well as depression	Android	50K+	4.4/5.0	Jun-19	Free(I)
		iOS	-	4.6/5.0	Dec-16	Free(I)
Woebot - Your Self-Care Expert*	A chatbot for guided CBT to manage stress and anxiety	Android	100K+	4.8/5.0	Nov-19	Free
		iOS	-	4.7/5.0	Nov-19	Free
Wysa: Mental Health Therapy*	A chatbot offering CBT and DBT techniques	Android	1M+	4.7/5.0	Nov-19	Free(I)
		iOS	-	4.7/5.0	Dec-19	Free(I)
Youper - Emotional Health*	A chatbot based on CBT and ACT techniques, monitoring and tracking mood changes	Android	1M+	4.7/5.0	Dec-19	Free(I)
		iOS	-	4.9/5.0	Dec-19	Free(I)

**Table 3:** Apps for hearing protection (Free = royalty free, Free(I) = in-app purchases, Free(A) = ad-supported, Free(I,A) = both). *Apps reported in literature.

App Name	Description	Platform	Users	Rating	Update	Pricing
Decibel X*	iOS equivalent of SPL Meter and Sound Meter	iOS	-	4.6/5.0	Jan-19	Free(I)
**dbTrack**	Uses earphones to measure sound exposure inside the ear canal	Android	10+	-	May-19	Free
**Hearangel**	Monitors music levels, notifies extreme and dangerous sound levels	Android	1000+	5.0/5.0	Sep-18	Free
iHEARu Here*	Crowdsourcing tool to report noise levels and find low sound exposure places	Android	10000+	3.1/5.0	Aug-18	Free
		iOS	-	-	Sep-18	Free
NIOSH Sound Level Meter*	Notifies user of current sound environment	iOS	-	4.7/5.0	May-19	Free
**Noise Control**	Measures surrounding sounds, allows recording and playback	Android	-	-	Sep-12	Free
		iOS	-	-	May-15	Free
NoiseCapture*	Evaluates noise environment and reports exposure	Android	100K+	4.4/5.0	Mar-19	Free
NoiSee*	Offers ANSI- or IEC-compliant sound level monitoring	iOS	-	4.6/5.0	Jan-19	0.89€(I)
NoiseScore*	Documents and visualizes environmental soundscape of users	Android	100+	4.5/5.0	Apr-18	Free
		iOS	-	4.5/5.0	Apr-18	Free
Soundcheck*	Identifies overexposing sounds and recommends hearing protection	Android	10K+	3.5/5.0	Jun-15	Free
		iOS	-	3.1/5.0	Jul-19	Free
Sound Meter*	Measures loudness of the environment, reference sound comparison	Android	10M+	4.6/5.0	Apr-19	Free(A)
Sound Meter - SPL Meter*	Sound Pressure Level (SPL) meter, reference sound comparison	Android	50K+	4.6/5.0	May-19	Free(A)
SoundPrint*	Crowdsourcing-based approach to find quiet places	Android	1000+	2.7/5.0	Apr-19	Free
		iOS	-	4.1/5.0	Apr-19	Free
SPLnFFT Noise Meter*	SPL with frequency analyzer, signal generator, dosimeter, etc.	iOS	-	4.7/5.0	Oct-18	3.59(I)
Too Noisy Pro*	Monitors noise levels in a closed environment, e.g., in classroom	Android	1000+	3.5/5.0	Feb-16	5.49€
		iOS	-	4.1/5.0	Jan-17	4.46€

**Table 4:** Apps for hearing testing (Free = royalty free, Free(I) = in-app purchases, Free(A) = ad-supported, Free(I,A) = both) *Apps reported in literature.

App Name	Description	Platform	Users	Rating	Update	Pricing
Audicus Hearing Test*	Quick hearing test at different frequencies	iOS	-	4.1/5.0	Oct-18	Free
**Better Hearing**	Hearing test to identify inaudible frequencies	iOS	-	2.3/5.0	Sep-12	Free(I)
Hearing Test*	Hearing test in normal and noisy environments shows results in audiogram	Android	1M+	4.4/5.0	Dec-16	Free
		iOS	-	2.8/5.0	Aug-13	Free(I)
Hearing Test Pro*	Paid version of Hearing Test	Android	1K+	4.6/5.0	Dec-16	3.58€
hearWHO*	Hearing test using headphones from WHO	Android	10K+	4.1/5.0	Mar-16	Free
		iOS	-	4.0/5.0	May-13	Free
Jacoti Hearing Center*	Helps in tracking hearing and provides results using DuoToneTM technology	iOS	-	3.8/5.0	Mar-19	Free
Mimi Hearing Test*	Determines hearing age based on hearing test	Android	10K+	3.0/5.0	Sep-18	Free
		iOS	-	4.6/5.0	Jan-19	Free
Signia Hearing Test*	Hearing test to identify words in background noise	Android	10K+	3.0/5.0	Nov-18	Free
		iOS	-	2.4/5.0	Jan-19	Free
Sound Scouts*	A game-based hearing test for children	Android	1K+	5.0/5.0	Mar-19	Free
		iOS	-	5.0/5.0	Mar-19	Free
Soundcheck*	Screens hearing and shows results in easy to read format	Android	10K+	3.5/5.0	Jun-15	Free
		iOS	-	2.9/5.0	Jul-15	Free
**Tone Generator**	Compares hearing with friends and family using customised frequency tones	iOS	-	3.8/5.0	Sep-16	Free(I)
Track Your Hearing*	Helps in monitoring and keeping track of hearing loss	Android	50+	1.0/5.0	Feb-18	Free
		iOS	-	-	Feb-15	Free
uHear*	Hearing test in normal and noisy environments	iOS	-	3.5/5.0	Oct-15	Free

**Table 5:** Apps for hearing enhancement (Free = royalty free, Free(I) = in-app purchases, Free(A) = ad-supported, Free(I,A) = both) *Apps reported in literature.

App Name	Description	Platform	Users	Rating	Update	Pricing
AUD-1*	Improves sound clarity using signal processing techniques	iOS	-	1.8/5.0	Nov-15	6.26€
BioAid*	Offers different amplifications for a personalized selection	iOS	-	2.3/5.0	Feb-15	Free
**Ear Agent**	Sound enhancements using headphones	Android	5M+	3.7/5.0	Feb-19	Free(I,A)
**Ear Booster**	Sound amplification using headphones	Android	100K+	3.8/5.0	May-19	Free(A)
EarMachine*	Sound enhancing app by recording via microphones	iOS	-	3.7/5.0	Jan-17	Free
**Ear Spy Pro**	Noise reduction and sound enhancement	Android	50K+	4.2/5.0	Dec-18	Free(A)
**EasyHearingAid**	Hearing impairment assistance using different frequencies	iOS	-	2.0/5.0	Jan-15	0.89€
**Hear**	Hearing enhancement and noise control with auto personalized adjustments	iOS	-	3.7/5.0	Feb-18	Free(I)
**Hear Coach**	An app to increase and improve listening abilities using a game	Android	5K+	4.2/5.0	Mar-16	Free
		iOS	-	2.6/5.0	Nov-19	Free
**Hearing Aid**	An app to record conversation and remove background noise	iOS	-	-	Apr-19	Free
Hearing Amplifier*	Enhances microphone input and outputs to headphones	iOS	-	2.3/5.0	Jan-19	Free
HearYouNow*	Sound amplifier for each ear	iOS	-	3.0/5.0	-	Free
Jacoti ListenApp*	Enables apple ear phone to improve sound clarity	iOS	-	3.1/5.0	Mar-19	Free
Petralex Hearing Aid*	Incoming sound enhancement	Android	100K+	3.9/5.0	Apr-19	Free(I)
		iOS	-	4.3/5.0	May-19	Free(I)
uSound*	Amplifies sounds based on profile created using hearing tests	Android	100K+	3.7/5.0	Jan-19	Free(I)
		iOS	-	3.7/5.0	Sep-18	Free(I)

**Table 6:** Apps for EEG recording (Free = royalty free, Free(I) = in-app purchases, Free(A) = ad-supported, Free(I,A) = both).

App Name	Description	EEG System	Platform	Users	Rating	Update	Pricing
**BrainLog**	Records brain activity, exports as .csv and stores in iCloud	Muse	iOS	-	1.8/5.0	Nov-15	1.09€
**EEG 101**	Teaches users the basics of EEG while displaying their own brain data	Muse brain sensing headband	Android	10K+	3.8/5.0	May-18	Free
**EEG Analyzer**	Brain activity recorded and stored as .csv in Dropbox	MindWave by NeuroSky	Android	10K+	4.0/5.0	Aug-14	1.79€
**eegID**	Captures electrical activity of brain and stores in .csv	MindWave by NeuroSky	Android	5K+	4.2/5.0	Mar-14	1.79€
**iBrainEEG2**	Brain activity analyzed in relation to functional connectivity network	Biosemi	Android	1K+	4.4/5.0	Mar-14	Free
**Muse Monitor**	Monitors EEG data and stores in .csv	Muse	Android	5K+	4.5/5.0	Jan-19	16.99€
		Muse	iOS	-	4.2/5.0	Jan-19	13.40€
**MyEmotiv**	Capture, save and playback recordings of brain activity in six “cognitive and emotional metrics”	EPOC	Android	5K+	2.5/5.0	Apr-19	Free
		EPOC	iOS	-	2.1/5.0	Apr-19	Free
**Persyst Mobile**	Mobile-phone–based EEG recorder	Persyst	iOS	-	1.8/5.0	Jul-18	Free
